# The relationship between perfectionism and marital outcomes: a systematic review and meta-analysis

**DOI:** 10.3389/fpsyg.2024.1456902

**Published:** 2024-12-10

**Authors:** Kiana Hadian Hamedani, Mohammad Reza Majzoobi, Simon Forstmeier

**Affiliations:** Department of Developmental Psychology and Clinical Psychology of the Lifespan, University of Siegen, Siegen, Germany

**Keywords:** perfectionism, marital outcomes, systematic review, meta-analysis, dyadic perfectionism

## Abstract

**Introduction:**

Perfectionism, as a transdiagnostic variable, can influence both the intrapersonal and interpersonal domains, one of the most significant of them is thought to be marital relationship. Given that perfectionism within a couple can negatively affect their intimate relationship and potentially lead to destructive outcomes, reviewing studies conducted in this area seems to be essential to gain a clearer understanding. Therefore, the present systematic review aims to examine the association between perfectionism and marital outcomes in married individuals.

**Methods:**

A comprehensive search was conducted across major scientific databases, including APA PsycArticles, PubMed, and Web of Science, using specific keywords and Boolean operators. Included were the English-Language studies published between 1980 and 2023 that investigated the relationship between perfectionism and marital outcomes in heterosexual couples. Out of the studies identified, 23 met the inclusion criteria for this review, of which 16 studies met the criteria for meta-analysis.

**Results:**

The meta-analysis indicated a small-to-moderate effect size for the association between perfectionism and marital outcomes (*r* = 0.26). Given the importance of marital relationship, such effect sizes for variables which may influence marital relationship bears significant value.

**Discussion:**

Therefore, the findings may encourage researchers to conduct various studies to examine specific details, moderators, and mediators in the relationship between perfectionism and marital outcomes and may also prompt couple therapists to address perfectionism as a destructive factor and integrate strategies into their protocols to reduce its impact in intimate relationships.

## Introduction

Marriage widely regarded as one of the most significant decisions in a person’s life, and marital satisfaction plays a crucial role in determining their overall quality of life and mental well-being. As a result, family psychologists often assess the quality of couples’ relationships based on their level of marital outcomes (Sevinç and Garip, 2010). Marital outcome in the current review refers to all the variables indicating the status of people’s marital relationship, among which marital satisfaction seems to be one of the most frequent. Although there are found many other marital outcomes such as marital quality, marital happiness, marital adjustment, marital conflict, etc., we focused on marital satisfaction, as the tip of the iceberg, in the introduction section.

Marital satisfaction is a situation in which the couples often feel happy (Mirgain and Cordova, 2007) and each partner has a positive assessment of their marital relationship (Ofovwe et al., 2013). Studies have shown that marital satisfaction is correlated with well-being variables in couples such as their mental health (Talayizadeh and Bakhtiyarpour, 2016), quality of life (Sohrabi et al., 2016), depression (Maroufizadeh et al., 2018), and alexithymia (Darjazini and Moradkhani, 2017).

Recent studies have identified several determining variables that play a crucial role in predicting couples’ marital satisfaction. These variables include personality factors (Claxton et al., 2012), emotional intelligence, hope and happiness (Anhange et al., 2017), love style (Odilavadze et al., 2019), dysfunctional relationship beliefs (Hamamci, 2005), and differentiation of self (Peleg, 2008) as well as stress, dyadic coping (Rusu et al., 2020). Notably, perfectionism and dyadic perfectionism have been highlighted as significant variables in predicting marital satisfaction.

Perfectionism characterized by striving for perfection and setting high standards for performance and Perfectionists tend to make highly critical evaluations of their own behavior and are hypersensitive to mistakes (Rice and Preusser, 2002). Perfectionists set high and often unreasonable standards for themselves and place a significant value on their efforts to achieve these standards, even in the face of difficulties (Slade and Owens, 1998).

Dyadic perfectionism is defined as a kind of other-oriented perfectionism, which are the perfectionism attitudes that people have about their romantic partners (Lopez et al., 2011). Confirmatory analysis shows that dyadic perfectionism consists of three factors. These factors are (1) order, determined by judgments about the punctuality of a partner; (2) high standards, determined by setting high-performance expectations, such as high expectations at work or at school from one’s partner; and (3) the discrepancy, characterized by the difference between the ideal standards expected from the partner and the perceived performance of the partner (Slaney et al., 2006).

Dyadic perfectionism can be conceptualized in two forms: compatible and incompatible. Compatible dyadic perfectionism is characterized by having high performance expectations from one’s partner, while incompatible dyadic perfectionism involves holding high performance expectations from one’s partner but constantly perceiving them as failing to meet these lofty expectations (Lopez et al., 2011). Research findings demonstrate that dyadic perfectionism in students’ romantic relationships places pressure on their partners and has a negative impact on the perceived quality of the relationship, overall satisfaction, and long-term commitment (Stoeber, 2012). Dyadic perfectionism is considered a distinct aspect of perfectionist concerns or efforts, as it involves having unrealistically high standards for others and meticulously evaluating their performance (Hewitt and Flett, 1991). Moreover, it is uniquely associated with antisocial and narcissistic personality traits (Sherry et al., 2016). Furthermore, dyadic perfectionism has been linked to dysfunctional interpersonal traits, including conflict, apathy, and hostility (Stoeber, 2014). Several studies have provided solid evidence supporting the negative relationship between dyadic perfectionism, the quality of the relationship (Mackinnon et al., 2012), and relationship satisfaction (Stoeber, 2012).

Notwithstanding a large number of studies which have been conducted regarding the correlation of perfectionism with marital outcomes, we find it hard to represent a clear picture of the correlation. Therefore, the existing gap shows the necessity of undertaking a systematic review to present an in-depth and precise picture in the area of inquiry. Moreover, the presence of the transparent picture would bring the researchers and therapists in the fields of marital outcomes and perfectionism to conduct further studies, to create therapeutic protocols, to undertake trial studies and to improve the existing therapeutic methods. For these reasons, we are going to carry out a systematic review to probe the relationship between perfectionism and dyadic perfectionism with marital outcomes. We represented our research question based on PICO framework. PICO stands for participant/population, intervention/indicator, comparator and outcomes that constitute an adequate research question (Schardt et al., 2007). The research question of this systematic review is how marital outcomes are associated with perfectionism and dyadic perfectionism. Our objectives in the current systematic review was to synthesize the existing research regarding the relationship between perfectionism and marital outcomes, as well as to provide an effect size for the mentioned relationship.

## Methods

### Pre-registration of review protocol

The protocol related to the current systematic review has been registered on the Open Science Framework (OSF) website (doi: 10.17605/OSF.IO/R3B5K).

### Search strategy

In order to find eligible studies for inclusion in this systematic review, we conducted searches in various scientific databases using our search query, including APA, PsycArticles, Psychology and Behavioral Sciences Collection, PSYNDEX Literature with PSYNDEX Tests, ProQuest (Social Sciences), ScienceDirect, Wiley Online Library, PubMed, OVID, and Web of Science. Our search formula was as follows [(“perfection*”AND (“marital” OR “couple” OR “relationship” OR “close relationship” OR “romantic” OR “marriage” OR “partner” OR “Partnership” OR “husband” OR “wife” OR “spouse” OR “dyad” OR “sexual satisfaction” OR “intimate relationships” OR “romantic idealization” OR “relationship beliefs” OR “interpersonal perception”))] from January, 2023 to April, 2023. The titles of articles retrieved using this search query were examined by the first author to determine whether they met the necessary criteria for inclusion in the detailed screening phase or not. Then, the selected articles in this phase were reevaluated by the second author and an expert in the field to ensure that they meet the precise screening criteria based on their titles and abstracts. Finally, the remaining articles, eligible for screening based on their titles and abstracts, were uploaded to the Eppi-Reviewer (Thomas et al., 2022) online software, where both authors could review the articles according to the predetermined inclusion criteria. In the first stage within the aforementioned software, the titles and abstracts of the articles were assessed, and in the second stage, the full text of the remaining articles was evaluated based on our inclusion and exclusion criteria to determine the final articles suitable for this systematic review. To ensure literature saturation, a manual search of the references of the remaining studies in the full-text assessment stage was performed, considering 6,500 references. It should be mentioned that we followed the PRISMA guidelines in conducting all parts of the current systematic review.

### Inclusion and exclusion criteria

**Figure 1:**
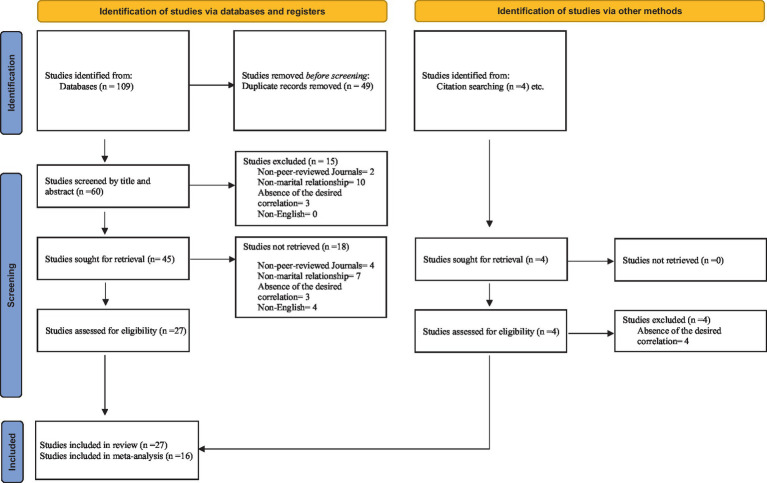
Flowchart of studies found through literature search and screening (Page et al., 2021).

We were seeking studies to include in this systematic review that generally investigate the relationship between perfectionism or dyadic perfectionism with marital outcomes, such as marital quality, satisfaction, adjustment, happiness, intimacy, conflict, disagreement, distress, etc. The inclusion criteria for studies in this systematic review had to (1) be in English, (2) be published in a peer-reviewed scientific journal between the years 1980 and 2023, and (3) examine couples in heterosexual relationships or those involved in long-term partnerships. Studies conducted on populations limited to same-sex couples were excluded. There were no restrictions on the geographical location of the studies. The process of selecting the studies included in this systematic review can be observed in Figure 1.

### Data extraction

After identifying the final studies that met the inclusion criteria for this systematic review, a data extraction form was designed by the authors to capture essential information from each article. The form first included sections for key theoretical insights from the introduction and discussion sections of the studies, which were crucial for providing a clear understanding of the research area and for inclusion in the introduction and discussion sections of the systematic review. This information included the scientific theories, conceptual models, and any mediating or moderating variables discussed in the studies. The subsequent sections of the data extraction form covered the following: the author, year and country of publication, study design, description of participants (i.e., sample size, age or age range, gender distribution, relationship status, ethnicity/origin, education level, and marital/relationship length), perfectionism measure, marital measure, key outcomes (effect size, if available). The studies were thoroughly reviewed by all authors of this review, and the data extraction was carried out by one author while being meticulously checked for accuracy by two other authors to ensure correct data extraction. In the last section of the extraction form, the extracted studies were assessed for eligibility to be included in the meta-analysis. To this end, the results section was carefully examined to confirm whether a correlation coefficient could be calculated between perfectionism and a marital outcome. If a study was deemed ineligible for inclusion in the meta-analysis, the reasons were clearly documented. These reasons included, for example, the absence of a total score for either the perfectionism or marital outcome measure, or both, which prevented the extraction of valid data for the meta-analysis.

### Risk of bias

**Figure 2:**
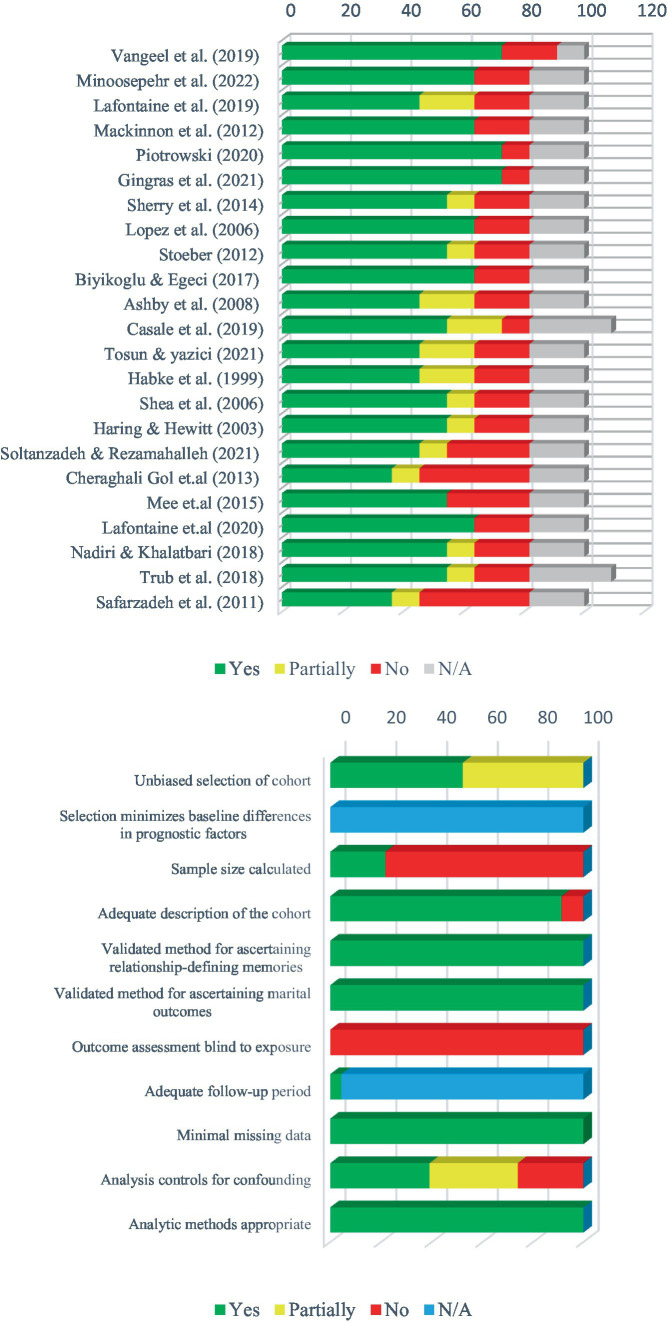
Risk of bias assessment in terms of studies as well as criteria presented in the ARHQ assessment tool.

To estimate the risk of bias in the studies included, each of these studies was considered by the first author (RS), the second author (SF) and another independent person expert in this field of inquiry. We also used the Agency for Research and Healthcare Quality Scale (AHRQ) as a tool to examine the bias of studies included. This measurement tool is compatible with various study designs (Forrester et al., 2017; Majzoobi and Forstmeier, 2022; Taylor et al., 2015; Williams et al., 2010). This tool contains 11 criteria for assessing the quality of studies, each of which also has several subscales, for important methodological factors of a study. The criteria of this scale included unbiased selection of the cohort, selection of the minimized baseline differences in prognostic factors, sample size calculated to be at 5% difference, adequate description of the cohort, validated method for ascertaining exposure, validated method for ascertaining clinical outcomes, outcome assessment blind to exposure, adequate follow-up period, completeness of follow-up, analysis controls for confounding, and appropriate analytic methods. The methodology and results of the input studies are reviewed according to the above criteria to determine whether they meet these criteria. Depending on whether they “fully,” “to some extent” and “not at all” meet the criteria, one of the words “No,” “Yes” and “Partially” is assigned to each study for each criterion. If a study does not meet the required quality or the necessary methodological criteria mentioned in this tool, it should be considered a probably biased article and excluded it from the review. Using this tool, we rated 11 criteria concerning each individual paper via “Yes,” “No,” “partial,” or “cannot tell” terms. This tool lacks a quantitative scoring system and focuses more on the qualitative description of bias or the quality of a study. Studies with a higher number of “Yes” ratings indicate greater quality and lower bias, whereas a higher number of “No” or “Partially” ratings reflects lower quality and higher bias. In this systematic review, it was determined that if a study received more than 5 “No” ratings out of the 11 criteria, it would be excluded due to insufficient quality or high bias. The results can be seen in Table 1. In addition, Figure 2 represents a visual representation of bias within and across studies.

**Table 1:** Risk of bias assessment of included studies based on the Agency for Research and Healthcare Quality assessment tool (Williams et al., 2010).

Authors	Unbiased selection of cohort	Selection minimizes baseline differences in prognostic factors	Sample size calculated	Adequate description of the cohort	Validated method for ascertaining DP^a^	Validated method for ascertaining MO^b^	Outcome assessment blind to exposure	Adequate follow-up period	Minimal missing data	Analysis controls for confounding	Analytic methods appropriate
Vangeel et al. (2020)	Yes	N/A	No	Yes	Yes	Yes	No	Yes	Yes	Yes	Yes
Minoosepehr et al. (2022)	Yes	N/A	Yes	Yes	Yes	Yes	No	N/A	Yes	No	Yes
Lafontaine et al. (2019)	partially	N/A	No	Yes	Yes	Yes	No	N/A	Yes	Partially	Yes
Mackinnon et al. (2012)	Yes	N/A	No	Yes	Yes	Yes	No	N/A	Yes	Yes	Yes
Piotrowski (2020)	Yes	N/A	Yes	Yes	Yes	Yes	No	N/A	Yes	Yes	Yes
Gingras et al. (2021)	Yes	N/A	Yes	Yes	Yes	Yes	No	N/A	Yes	Yes	Yes
Sherry et al. (2014)	Yes	N/A	No	Yes	Yes	Yes	No	N/A	Yes	Partially	Yes
Lopez et al. (2006)	Yes	N/A	No	Yes	Yes	Yes	No	N/A	Yes	Yes	Yes
Stoeber (2012)	Yes	N/A	No	Yes	Yes	Yes	No	N/A	Yes	Partially	Yes
Biyikoglu and Egeci (2017)	Yes	N/A	No	Yes	Yes	Yes	No	N/A	Yes	Yes	Yes
Ashby et al. (2008)	Partially	N/A	No	Yes	Yes	Yes	No	N/A	Yes	Partially	Yes
Casale et al. (2020)	Partially	N/A	Yes	Yes	Yes	Yes	No	N/A	Yes	Partially	Yes
Tosun and Yazici (2021)	Partially	N/A	No	Yes	Yes	Yes	No	N/A	Yes	Partially	Yes
Habke et al. (1999)	Partially	N/A	No	Yes	Yes	Yes	No	N/A	Yes	Partially	Yes
Shea et al. (2006)	Yes	N/A	No	Yes	Yes	Yes	No	N/A	Yes	Partially	Yes
Haring et al. (2003)	Partially	N/A	No	Yes	Yes	Yes	No	N/A	Yes	Yes	Yes
Soltanzadeh Rezamahalleh (2021)	Partially	N/A	No	Yes	Yes	Yes	No	N/A	Yes	No	Yes
Gol et al. (2013)	Partially	N/A	No	No	Yes	Yes	No	N/A	Yes	No	Yes
Mee et al. (2015)	Yes	N/A	No	Yes	Yes	Yes	No	N/A	Yes	No	Yes
Lafontaine et al. (2020)	Yes	N/A	No	Yes	Yes	Yes	No	N/A	Yes	Yes	Yes
Nadiri and Khalatbari (2018)	Partially	N/A	Yes	Yes	Yes	Yes	No	N/A	Yes	No	Yes
Trub et al. (2018)	Partially	N/A	No	Yes	Yes	Yes	No	N/A	Yes	Yes	Yes
Safarzadeh et al. (2011)	Partially	N/A	No	No	nYes	Yes	No	N/A	Yes	No	Yes

^aDyadic perfectionism. bmarital outcome.^

### Meta-analysis

Among all the studies included in this systematic review, 16 studies were eligible to be entered into a meta-analysis to investigate the relationship between perfectionism and marital outcomes. Some studies examined the mentioned relationship separately for men and women, and other studies explored the association of women’s perfectionism with male’s marital outcomes and vice versa. Additionally, in some studies, the total score for perfectionism was not given, and instead, subscale scores of this variable were reported. Therefore, the number of entries in the meta-analysis presented in this study was 70 cases. Meanwhile, missing data were found in seven studies (Mee et al., 2015; Gol et al., 2013; Haring et al., 2003; Habke et al., 1999; Ashby et al., 2008; Biyikoglu and Egeci, 2017; Stoeber, 2012), in which overall scores for the variables of perfectionism or marital satisfaction were not provided, and therefore, we were unable to include them in our meta-analysis. Pearson’s correlation coefficient was used to examine the effect size of the relationship between perfectionism and marital outcomes. Furthermore, the Cochran’s Q test and I^2^ index were used to assess heterogeneity of effect sizes. The reason for reporting the I^2^ index is that in meta-analyses with small sample sizes, it can provide more informative results than Q (Higgins et al., 2003).

One of the main assumptions of the meta-analysis is publication bias, which implies that studies should have effect sizes with a relatively homogeneous distribution around the mean line. In this systematic review, the funnel plot, Egger’s regression intercept, and Kendall’s S index were used to investigate publication bias. As the included studies in this systematic review came from different age, racial, and cultural groups, we used a random-effects model, following Hunter and Schmidt’s approach (2004, as cited in Field and Gillett, 2010), to report the overall effect size. Consequently, we can have higher confidence in generalizing the obtained effect size to the studies included in this systematic review and other studies conducted in this area (Field and Gillett, 2010). The provided meta-analysis in this study was conducted using the Comprehensive Meta-Analysis Software CAM-2.

## Results

### The characteristics of the studies included

The characteristics of the studies included in this systematic review are fully described in Table 2. Based on this, all the included studies were published between 1999 and 2022. Out of the total studies, five were conducted in the United States, five in Iran, seven in Canada, two in Turkey, and one study each in Pakistan, England, Italy, and Malaysia. Among the entered studies, 22 had a correctional design, and 2 had a longitudinal design. The descriptive nature of the design in most of the included studies allowed us to present more consistent and integrated results. Moreover, the uniformity in the study design facilitated the meta-analysis process. The study population examined in these studies included heterosexual couples from different countries, races, and educational levels. The participants’ age range was between 19.4 and 38.9 years, with the minimum and maximum duration of their relationship ranging from less than 1 year to 26 years, respectively. Additional information regarding the characteristics of the participants of studies included in the current systematic review can be found in Table 2.

**Table 2:** Description of included studies.

Author, year, country	Design	Description of participants	Perfectionism measure	Marital measure	Key outcome (effect size, if presented)
Vangeel et al. (2020), USA	Longitudinal	*n* = 161 (M_age_ = 28.91, SD = 1.68, range = 27–32); 55.9% female; 96.9% heterosexual; 90.6% lived together; M_RL_ = 7.29Y (SD = 4.21), 68.3% graduated/higher education	RBI (Eidelson and Epstein, 1982)	RAS (Hendrick et al., 1998)	Sig. relationship between sexual PERF and RS (*r* = −0.27, *β* = −0.37)
Minoosepehr et al. (2022), Iran	Correlational and Cross-sectional	*n* = 210 (129 female, 81 male); M _age_ = 36.81 M_RL_ = 11.20	MSPQ (Snell, 1995, 2011)	CBM (Pines, 1996)	Sig. relationship between Sexual PERF and marital burnout (*r* = 0.29, *β* =0.20)
Lafontaine et al. (2019), Canada	Correlational	*n* = 170 mixed-sex couples; 62% = common-law relationship and 19% = married; 12% = had children M_age_ = 30Y (range = 19–78Y); 84% = Caucasian; 60% = had a university degree	DCI (Bodenmann, 2008)	ECR (Brennan et al., 1998)	Sig. relationship between SORP M and OORP M with Dyadic Coping M (*r* = −0.11, *r* = −0.37; B = -, B = -0.18); Sig. relationship between SORP M and OORP M with Dyadic Coping W (*r* = −0.08, *r* = −0.22); Sig. relationship between SORP W and OORP W with Dyadic Coping W (*r* = −0.27, *r* = −0.49; B = -B = -0.33); Sig. relationship between SORP M and OORP with Dyadic Coping M (*r* = −0.15, *r* = −0.19).
Mackinnon et al. (2012), Canada	Longitudinal	*n* = 226 heterosexual (226 men, 226 women); Caucasian (men 88.5%, women 88.5%), Canadian-born (men 85.4%, women 88.5%), M_RL_ = 2.10Y (SD = 2.23), 38.9% = cohabiting	Perfectionistic concerns (Lopez et al., 2006) other-oriented PERF (Hewitt and Flett, 1991)	IQS (Oishi and Sullivan, 2006) RIB (Murray et al., 2003)	Relationship between men’s other-oriented PERF with men’s dyadic conflict (Murray *r* = 0.27, Oishi *r* = 0.29) and women’s dyadic conflict (Murray *r* = −0.05, Oishi *r* = 0.01); relationship between women’s other-oriented PERF with women’s dyadic conflict (Murray *r* = 0.11, Oishi *r* = 0.11) and men’s dyadic conflict (Murray, *r* = −0.08; Oishi, *r* = 0.07).
Piotrowski (2020), Poland	Correlational	*n* = 459 individuals (264 = female and 195 = men), (*M_age_* = 33.88, *SD =* 4.39), 78% = married, 22% = informal relationships; *M_RL_ =* 9.56 Y, median = 9.0, *SD =* 4.61	DAPS	IRD	Relationship between men’s POP standards, order, and discrepancy with men’s relationship stress (*r* = 0.45, 0.09, 0.76) and with men’s relationship conflicts (*r* = 0.40, 0.05, 0.73); relationship between women’s POP standards, order, discrepancy with Women’s relationship stress (*r* = 0.35, 0.16, 0.59) and with women’s relationship conflicts (*r* = 0.36, 0.13, 0.60)
Gingras et al. (2021), Canada	Cross-sectional	*n* = 80 French-Canadian couples; women’s M_age_ = 30.48 (SD = 3.72), men’s M_age_ = 31.09 (SD = 4.87); 36.3% married, 63.7% common law unions, M_RL_ = 7.55Y. elementary or high school (women: 12.5%, men: 21.3%), preuniversity (women: 13.8%, men: 20.0%), university degree (women: 73.8%, men: 58.8%)	DAS (Spanier, 1976)	PQ-R (Langlois et al., 2010)	Relationship between men’s adaptive PERF with men’s RS (*r* = 0.28) and women’s RS (*r* = 0.18); relationship between men’s maladaptive PERF with men’s RS (*r* = 0.03) and women’s RS (*r* = 0.1); relationship between women’s adaptive PERF with women’s RS (*r* = −0.03) and men’s RS (*r* = −0.16); relationship between women’s maladaptive PERF with women’s RS (*r* = −0.41) and men’s RS (*r* = −0.26)
Sherry et al. (2014), Canada	Cross-sectional	*n* = 226 heterosexual couples (226 men, 226 women); men’s M_age_ = 22.35Y (SD = 4.52); women’s M_age_ = 21.48Y (SD = 4.13); Caucasian (men 88.5%; women 88.5%), M_RL_ = 2.10Y (SD = 2.23)	SPP (Flett et al., 1991) and MPS (Hewitt and Flett, 1991)	DIRI (Joiner and Metalsky, 2001) daily conflict (Murray et al., 2003)	In men, wives’ expectations for them to be SPP were significantly correlated with both self-report DC (*r* = 0.29) and partner-report conflict (*r* = 0.33); wives’ belief that their husbands’ expect for them to be SPP were correlated significantly with husbands’ self-report DC (*r* = 0.29) and partner-report conflict (*r* = 0.37). Both kinds of self-report partner-specific SPP (*r* = 0.29) and partner-report SPP (*r* = 0.24) were associated with reassurance-seeking. In women, husbands’ expectations for them to be SPP were significantly correlated with both self-report DC (*r* = 0.16) and partner-report conflict (*r* = 0.28). Husbands’ belief that their wives’ expect for them to be SPP were not correlated significantly with wives’ self-report DC (*r* = 0/04) and partner-report conflict (*r* = 0.13). Both self-report partner-specific SPP (*r* = 0.23) and partner-report SPP (*r* = 0.20) were associated with reassurance-seeking.
Lopez et al. (2006), USA	Correlational	*n* = 121 college students (82 women, 39 men); M_age_ = 20.5Y (*SD =* 4.26); 88.4% predominantly single; 31% White, 21% Black, 28% Asian, 17% Hispanic, 3% Multiracial; M_RL_ = 22 M (*SD =* 34)	DAPS (Shea et al., 2006)	ECR (Brennan et al., 1998); RAS (Hendrick et al., 1998)	Sig. negative relationship between discrepancy and MS (*r* = −0.61), but not between standards (*r* = 0.07) and order (0.11) with MS at time 1; Discrepancy (*r* = −0.55) and standards (*r* = 0.26) were negatively related to MS, respectively. no Sig. relationship between order and MS at time 2 (*r* = 0.22).
Stoeber (2012), UK	Correlational	*n* = 116 (53 male, 63 female); 58 couples (53 heterosexual, 5 homosexual); M_age_ = 21.4Y (SD = 2.9), M_RL_ = 1.6Y (SD = 1.5)	MPS (Hewitt and Flett, 1991)	RAS (Hendrick et al., 1998); CI (Stanley and Markman, 1992)	Participants’ partner-oriented PERF had a positive effect on their partner’s partner-prescribed PERF and a negative effect on their own RS and long-term commitment. Participants’ partner-prescribed PERF also had a negative effect on their own RS.
Biyikoglu and Egeci (2017), Turkey	Correlational	*n* = 290, 188 women (64.8%) and 102 men (35.2%); M_age_ = 38.97 (SD = 9.48). M_RL_ = 14.52 Y (SD = 9.48). 23.1% primary/elementary school (*n* = 67), 28.3% high school (*n* = 82), and 48.6% higher education (*n* = 141) (academy, university and graduate level).	MPS (Hewitt and Flett, 1991)	DAS (Spanier, 1976)	Individuals who had self-oriented PERF reported greater level of marital adjustment. Specifically, socially-prescribed PERF in males and self-oriented PERF in females were found to be the determinant figure on the issue of marital adjustment.
Ashby et al. (2008), USA	Correlational	*n* = 197 couples; Men’s M_age_ *=* 27.47 (SD = 5.07), Women’s M_age_ = 25.61 (SD *=* 4.36); 90% White/European American, 4% African American, 3% Asian American, 2% Latino/Latina, 1% other	APS-R (Slaney et al., 2001)	PREPARE (Olson et al., 1987)	Couples in which both partners were adaptive perfectionists tended to cluster in more functional couple types. Maladaptive PERF in one partner somewhat decreased the likelihood of higher quality relationships, except in the case of matches with a non-perfectionist.
Casale et al. (2020), Italy	Correlational	*n* = 356 couples; M_age_ = 26.18Y (SD = 7.47), M _RL_ = 5.1Y (SD = 6.56); 60 married couples (17.44%); M_RL_ = 15.05Y (SD = 11.41). 100% Caucasian	PSPS (Hewitt et al., 2003)	SS (Busby and Gardner, 2008)	Sig. negative relationship between men’s PERF and their own MS (*r* = −0.19), the same does not hold for the men’s PERF and women’s MS (*r* = −0.5). women’s PERF was significantly related to women’s MS (*r* = −0.21), but not to men’s MS (*r* = −0.07).
Tosun and Yazici (2021)	Correlational	*n* = 246 college students (70.7% female, 24.8% male, 4.5% unspecified). M_RL_ = 31.47 months (±32.13); M_age_ = 22.76 (±3.52).	DAPS (Slaney et al., 2006)	RQS (Pierce et al., 1991)	Discrepancy (a subscale of dyadic PERF) was significantly related to subscales of relationship quality, namely social support (*r* = −0.36) depth (*r* = −0.37) and conflict (*r* = 0.45). High standards were significantly related to conflict (*r* = 0.27) and not to social support (*r* = −0.07) and depth (*r* = −0.01). The subscale of order was not significantly related to the subscale of social support (*r* = 0.13) and not to depth (*r* = 0.07) and conflict (*r* = 0.12).
Habke et al. (1999), Canada	Correlational	*n* = 82 couples; Men’s M_age_ = 29.6 (SD = 7.41), women’s M_age_ = 27.08 (SD = 6.4); 39 married (53%), 35 common-law (47%), M_RL_ = 26.7 months (SD = 11.45)	PSPS (Hewitt, et al., 1996); MPS (Hewitt and Flett, 1991)	PSSI (Pinney et al., 1987); DAS (Spanier, 1976)	The results showed that the interpersonal dimensions of trait PERF were negatively related to general sexual satisfaction and sexual satisfaction with the partner for both husbands and wives.
Shea et al. (2006), USA	Correlational	*n* = 389 students; 294 Mid-Atlantic sample [199 women, 95 men; M_age_ = 20.33 (SD *=* 3.4 1)], 99 Midwest sample [62 women, 33 men; M_age_ = 25.56 (SD = 6.28)]; 90.8% European American, 2.1% African American, 1.8% Latino/Latina American, 1.3% Asian American, 0.3% Native American, 2.6% international students, 1% other; M_RL_ = 13.68 M	APS-R (Slaney et al., 2001); Self-Oriented PERF	RAS (Hendrick et al., 1998)	Discrepancy (subscale of PERF) was significantly related to avoidant (*r* = 0.42) and anxiety (*r* = 0.37) in relationship in women. Two subscales of high standards and order were not significantly related to avoidant (*r* = −0.05; *r* = −0.02) and anxiety (*r* = 0.08; *r* = −0.03) in relationship in women. Sig. relationship between subscale of discrepancy and avoidant in relationship in men. No Sig. relationship between avoidant in intimate relationship and high standards (*r* = −0.17) and order (*r* = −0.01), and between anxiety in relationship and high standards (*r* = −0.15), discrepancy (*r* = 0.04) and order (*r* = −0.06) in men.
Haring et al. (2003), Canada	Correlational	*n* = 67 couples; M = 26.6 M (SD *=* 11.4); Men’s M_age_ *=* 30.6 (SD = 10.8); women’s M_age_ *=* 27.4 (SD *=* 6.6); 129 Whites (84.9%), 4 Asians (2.6%), 1 Native American (0.6%), and 18 not specify (11.8%); M_edu_ = 13.62Y; 39 married (51%), 37 common law spouses (49%)	MPS (Hewitt and Flett, 1991)	DAS (Spanier, 1976); MHS (Azrin et al., 1973); ARI (Schaefer and Burnett, 1987)	Sig. relationship of Husbands’ marital adjustment and husbands’ socially prescribed PERF (*r* = −0.39) and no Sig. relationship of husbands’ self-oriented (*r* = −0.07) and other-oriented (*r* = −0.13) PERF; husbands’ marital functioning had Sig. negative relationship with husbands’ socially prescribed PERF (*r* = −0.57) and had no Sig. relationship with husbands’ self-oriented (*r* = −0.17) and other-oriented (*r* = −0.11) PERF; husbands’ marital happiness had no Sig. relationship with husbands’ socially prescribed (*r* = −0.11), self-oriented (*r* = 0.00) and other-oriented PERF (*r* = −0.04). 4. Husbands’ marital adjustment (*r* = −0.41), functioning (*r* = −0.42) and happiness (*r* = −0.30) were correlated significantly with wives’ socially prescribed PERF. Husbands’ marital adjustment (*r* = 0.01; *r* = −0.08), functioning (*r* = −0.11; *r* = −0.15) and happiness (*r* = 0.01; *r* = −0.07) were not associated significantly with wives’ self-oriented and other-oriented PERF. 5. wives’ marital adjustment (*r* = −0.57), functioning (*r* = −0.63) and happiness (*r* = −0.44) were significantly related to wives’ socially prescribed PERF besides, wives’ marital adjustment (*r* = −0.31) and happiness (*r* = −0.25) were significantly correlated with other-oriented PERF, but the same does not hold for marital functioning (*r* = −0.15). Wives marital adjustment (*r* = −0.07) functioning (*r* = −0.17) and happiness (*r* = −0.08) were not significantly related to self-oriented PERF. 6. wives’ marital adjustment (*r* = −0.22), functioning (*r* = −0.25), and happiness (*r* = −20) were significantly related to husbands’ socially prescribed PERF. However, wives’ marital adjustment (*r* = 0.04; *r* = −0.01), functioning (*r* = −0.04; *r* = −0.01) and happiness (*r* = 0.08; *r* = 0.09) were not significantly correlated to husband’s self-oriented and other-oriented PERF.
Soltanzadeh Rezamahalleh (2021), Iran	Correlational	*n* = 369 (51% men, 49% women); Age = 26.8% under 25 Y, 43.6% between 25–30 Y, 29.6% over 30Y; 3.35% bachelors, 2.56% Master, 5.8% PhD	PERF (Owens Glynn et al., 1995)	AIQ (Watley, 2008)	Positive PERF was found to be associated negatively to marital infidelity (*r* = −0.403). Besides, negative PERF was positively related to marital infidelity (*r* = 0.433).
Gol et al. (2013), Iran	Correlational	*n* = 123 (67 men, 56 women)	PERF (Owens Glynn et al., 1995)	EMSQ (Fowers and Olson, 1993)	Positive and negative PERF are able to predict MS (R^2^ = 0.141).
Mee et al. (2015), Malaysia	Correlational	*n* = 30 graduate students; M_age_ = 34.52 (SD = 6.63); 21 females (70%); 9 males (30%); 27 Malay (90%), 2 Indian (6.7%), 1Chinese (3.3%); 21 master students (70%), 9 PhD students (30%)	DAPS (Shea et al., 2006)	EMSQ (Fowers and Olson, 1993)	There was no Sig. relationship between two components of self-PERF namely self-high standards (*r* = −0.12) and self-discrepancy (*r* = −0.12) with MS. There was a Sig. relationship between two components of dyadic –PERF namely dyadic high standards (*r* = −0.50) and dyadic discrepancy (*r* = − 0.50).
Lafontaine et al. (2020), Canada	Correlational	*n* = 564 university students; 422 females (74.8%),142 males (25.2%); M_age_ = 19.40 (SD = 1.79); M_RL_ = 54 (9.6%) <1Y, 387 (68.6%) 1-2Y, 112 (19.9%) 3-5Y, 9 (1.7%) 6-10Y, 2 (0.4%) >10Y; 11 (2.0%) married, 482 (85.5%) intimate partner	MPS (Cox et al. 2002)	PCS (Brassard and Lussier, 2007)	Self-oriented romantic PERF was positively related to marital conflict (*r* = 0.17), psychological IPV (*r* = 0.10) and physical IPV (*r* = 0.08). Besides, socially-oriented romantic PERF was positively related to marital conflict (*r* = 0.39), psychological IPV (*r* = 0.18) and physical IPV (*r* = 0.16).
Nadiri and Khalatbari (2018), Iran	Correlational	*n* = 252 married students (142 female, 110 male); 78 bachelor, 105 Master, 69 PhD	PERF (Owens Glynn et al., 1995)	EMSQ (Fowers and Olson, 1993)	There was a Sig. negative relationship between PERF and MS (*r* = −0.62)
Trub et al. (2018), USA	Correlational	*n* = 382, M_age_ = 38.7 (22–86, SD *=* 12.7); M_RL_ = 13.1Y (SD *=* 11.9); 71.7% women, 28.3% men; 89.5% White, 3.1% Hispanic, 2.6% Asian, 1.3% African-American, 0.8% multiracial, 2.6% other; 96.6% heterosexual; 61.5% Graduate, 32.2% undergraduate degree, 5% college, 1.3% high school graduates	MPS (Hewitt and Flett, 1991)	Revised DAS (Busby et al., 1995)	There was a Sig. negative relationship between other-oriented PERF (*r* = 0.143) and socially-oriented PERF (*r* = 0.400) with RS. However, the same does not hold for self-oriented PERF (*r* = − 0.036)
Safarzadeh et al. (2011), Iran	Correlational	*n* = 200 students (100 female, 100 male)	APS	EMSQ (Fowers and Olson, 1993)	There was a Sig. negative relationship between perfectionism and MS (*r* = − 0.202)

AIQ, attitude to infidelity questionnaire; APS, Ahvaz Perfection Scale; APS-R, almost perfect scale-revised; ARI, autonomy and relatedness inventory; CBM, couple burnout measurement; CI, commitment inventory; DAPS, dyadic almost perfect scale; DAS, dyadic adjustment scale; DC, daily conflict; DCI, dyadic coping inventory; DIRI, depressive interpersonal relationships inventory; ECR, experiences in close relationships; EMSQ, enrich marital satisfaction questionnaire; IQS, interpersonal qualities scale; IRD, Intimate relationship difficulties; MHS, marital happiness scale; MPS, multidimensional perfectionism scale; MS, marital satisfaction; MSPQ, multidimensional sexual perfectionism questionnaire; PCS, perception of conflict scale; PERF, perfectionism; PQ-R, perfectionism questionnaire-revised; PREPARE, pre-marital personal and relationship evaluation; PSPS, perfectionistic self-presentation scale; PSSI, Pinney sexual satisfaction inventory; RAS, relationship assessment scale; RBI, relationship belief inventory; RIB, rejecting interpersonal behaviors; RQS, relationship quality scale; RS, relationship satisfaction; Sig., significant; SS, satisfaction scale; SPP, socially prescribed perfectionism; Y, year.

### Risk of bias assessment

Table 1 provides a complete description of the bias assessment of the studies included in this systematic review. The results reported in this table are obtained from examining the characteristics of the studies included based on the criteria presented in the Assessment of Multiple Systematic Reviews (ARHQ) scale. Besides, in Figure 2, the degree of bias of each study based on ARHQ is shown graphically. The first graph in Figure 2 shows the degree of bias of each study, and the second graph shows the results of the overall quality score of the studies included for each of the 11 criteria in the ARHQ.

Based on the conducted evaluation, it seems that most of the criteria of the ARHQ have been observed in the conducted studies. All 23 studies included in this review met the required quality standards according to this criterion. However, the criterion “Outcome assessment blinded to exposure” was not adhered to in the majority of the studies, which could somewhat reduce the quality of the studies and increase the risk of type I errors. Additionally, the criterion “Sample size calculated” was observed in less than half of the included studies, which may increase the risk of type II errors. Further information about the bias level of each study can be found in Table 1 and Figure 2.

### Measures used in the included studies

All studies included in this systematic review had used valid and standardized questionnaires validated in the countries in which the studies were conducted. To measure perfectionism in relationship, six studies used Multidimensional Perfectionism Scale (MPS) (Hewitt and Flett, 1991), four studies used Dyadic Almost Perfect Scale (DAPS) (Shea et al., 2006), two studies used Almost Perfect Scale-Revised (APS-R) (Slaney et al., 2001) and two studies used Perfectionistic Self-Presentation Scale (PSPS) (Hewitt et al., 2003). To measure marital outcomes, four studies applied Enrich Marital Satisfaction Scale (EMSQ) (Fowers and Olson, 1993), four studies applied Relationship Assessment Scale RAS (Hendrick et al., 1998), and three studies applied Dyadic adjustment scale (DAS) (Spanier, 1976). The questionnaires used in the studies included in the current systematic review are presented in Table 2.

### The relationship between perfectionism and marital outcomes

Considering all found articles, the most important consequence of perfectionism in couples seems to be marital satisfaction. As a result, most of the studies reviewed above showed a negative correlation between perfectionism and marital satisfaction (Casale et al., 2020; Gingras et al., 2021; Gol et al., 2013; Lopez et al., 2006; Mee et al., 2015; Nadiri and Khalatbari, 2018; Safarzadeh et al., 2011; Casale et al., 2020; Stoeber, 2012; Trub et al., 2018; Vangeel et al., 2020). Marital conflict is another common consequence of perfectionism in couples (Lafontaine et al., 2020; Mackinnon et al., 2012; Piotrowski, 2020; Tosun and Yazici, 2021). Therefore, it can be said that after marital satisfaction, marital conflict can be another consequence of perfectionism in couples.

Other consequences of perfectionism in couples include marital adjustment (Biyikoglu and Egeci, 2017; Haring et al., 2003), quality relationships (Ashby et al., 2008; Tosun and Yazici, 2021), marital burnout (Minoosepehr et al., 2022), dyadic coping (Lafontaine et al., 2019), relationship stress (Piotrowski, 2020), reassurance-seeking (Sherry et al., 2014), long-term commitment (Stoeber, 2012), general sexual satisfaction and sexual satisfaction with the partner (Habke et al., 1999), marital infidelity (Soltanzadeh Rezamahalleh, 2021), psychological and physical IPV (Lafontaine et al., 2020), avoidant and anxiety (Shea et al., 2006) and marital functioning and happiness (Haring et al., 2003).

### Meta-analysis

**Figure 3:**
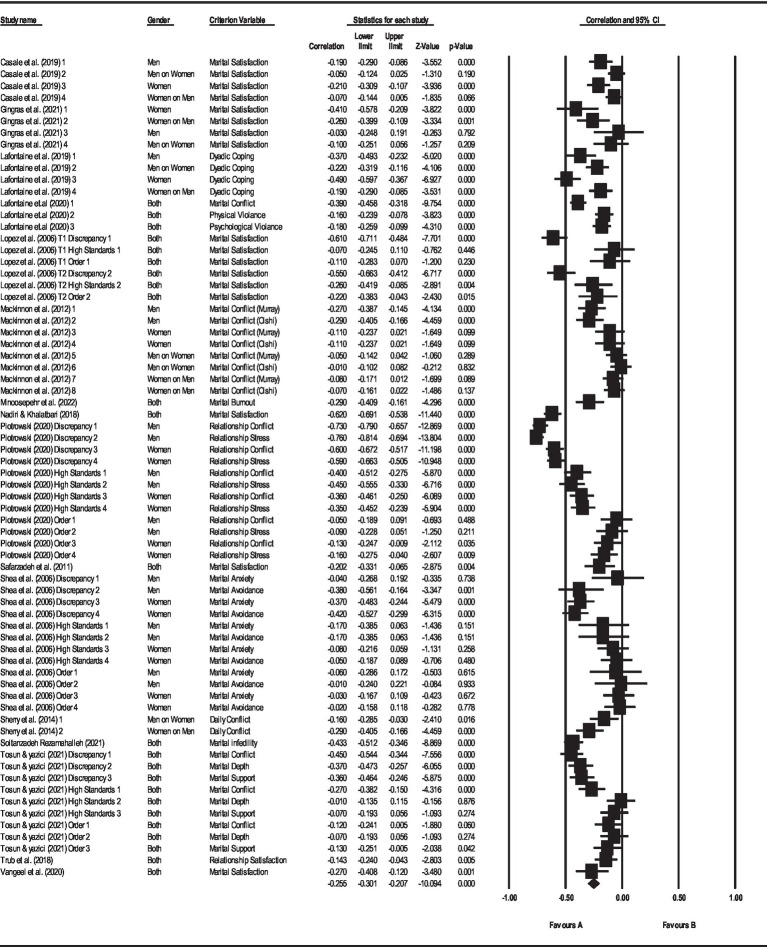
The result of meta-analysis for the relationship between perfectionism and Marital outcomes.

The effect size obtained for the relationship between perfectionism and marital outcomes was *r* = −0.26 (Figure 3), which, according to Cohen’s (1988) effect size table, falls into the category of a small-to-moderate effect size.

**Figure 4:**
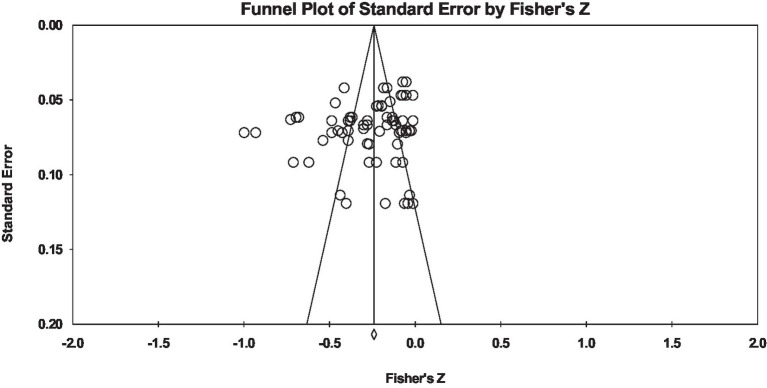
Funnel Plot for the assessment of the publication bias of the included studies.

Additionally, the funnel plot presented in Figure 4 to assess the publication bias of the included studies in this meta-analysis shows that, except for a few limited studies, the remaining studies are symmetrically distributed on both sides of the effect size line within the triangle. In other words, no study excessively deviated from the effect size line in this meta-analysis. The Kendall’s tau value was −0.12, which was significant in both one-tailed tests (*p* = 0.058) and two-tailed tests (*p* = 0.11). Furthermore, the Egger’s regression intercept value was −2.62, which was significant in both one-tailed tests (*p* = 0.052) and two-tailed tests (*p* = 0.085). Moreover, Q and I^2^ tests were used to assess the homogeneity of effect sizes. Based on the obtained results, the Q value for the relationship between perfectionism and marital outcomes was 328.761 with 69 degrees of freedom, which was significant (*p* < 0.001), indicating homogeneity of effect sizes of the included studies in this systematic review. The I^2^ statistic was 90.937.

## Discussion

This systematic review including a meta-analysis was conducted to investigate the relationship between perfectionism and marital outcomes in married individuals. The consideration of the studies included illustrated that perfectionism is one of the effective variables in predicting marital outcomes such as marital quality, satisfaction, adjustment, happiness, intimacy, conflict, disagreement, distress, etc. These findings were also confirmed in our meta-analyses in which it was found that perfectionism was associated with marital outcomes (*r* = 0.26). The results obtained in this meta-analysis are consistent with the findings of the studies included in this systematic review as well as with other literature in this field. Specifically, these studies also had found a negative association between perfectionism and positive marital outcomes, and a positive association with negative marital outcomes. Although several mediating variables were identified in the studies included in the systematic review, a meta-analysis could not be performed on them due to the lack of repeated instances of any single variable across multiple studies.

### The synthesis of the studies in this systematic review

In synthesizing the studies on perfectionism and marital outcomes reviewed in the current systematic review, the majority of studies consistently point to a negative correlation between perfectionism, particularly maladaptive forms, and various dimensions of marital satisfaction and stability. Studies included in this review indicate that perfectionism is often associated with lower marital satisfaction and increased marital conflict. For instance, several studies identified maladaptive perfectionism as a significant predictor of marital dissatisfaction, where perfectionistic concerns about meeting high standards or avoiding mistakes resulted in heightened marital stress and conflict.

In contrast, a few studies highlight that adaptive forms of perfectionism, characterized by realistic goal-setting and self-improvement, may contribute positively to marital satisfaction when partners perceive perfectionistic tendencies as motivational rather than critical. For instance, research by Haring et al. (2003) demonstrated that adaptive perfectionism, which fosters personal development without overly stringent standards, was associated with better marital functioning.

Furthermore, dyadic perfectionism, perfectionistic expectations directed toward a partner, has been shown to uniquely contribute to relationship difficulties. Studies such as those by Stoeber (2012) and Mackinnon et al. (2012) underscore that when individuals hold their partners to unattainable standards, this “other-oriented” perfectionism tends to correlate with higher levels of conflict, diminished relational satisfaction, and even marital burnout in extreme cases.

The findings across these studies converge in emphasizing that perfectionism, particularly in its maladaptive forms, undermines marital outcomes by promoting unrealistic expectations and dissatisfaction. However, divergence in findings regarding adaptive perfectionism suggests a complex interplay between motivational factors and relational perceptions. The synthesis of these studies not only underscores perfectionism’s direct effect on marital outcomes but also highlights its potential mediators.

### The potential mediators between perfectionism and marital outcomes

Among the studies included in this systematic review, few have examined mediating variables in the relationship between perfectionism and marital outcomes. Minoosepehr et al. (2022) demonstrated that a negative sexual self-concept significantly mediates the relationship between sexual perfectionism and marital burnout. Similarly, Haring et al. (2003) found that the use of negative coping styles serves as a significant mediator between social perfectionism and marital functioning. Mackinnon et al. (2012) also identified dyadic conflict as a significant mediator in the link between perfectionism and depression within marital relationships. Furthermore, marital perfectionism itself has been shown to play a mediating role in other relational contexts. Lafontaine et al. (2020) reported that romantic perfectionism mediates the association between insecure attachment and intimate partner violence, while Lafontaine et al. (2019) found that perfectionism significantly mediates the relationship between insecure attachment and dyadic coping. These findings highlight the multifaceted role of perfectionism both as a direct predictor and as a mediator that influences various relational outcomes, particularly in the context of insecure attachment and marital satisfaction.

### The demographic variables in the relationship between perfectionism and marital outcomes

To better understand the relationship between perfectionism and marital outcomes, it is essential to consider contextual factors such as cultural background, socioeconomic status, and demographic variables. It seems that different cultural values, for instance, influence how perfectionism manifests within relationships. It is likely that cultures that emphasize high performance and achievement may intensify perfectionist tendencies, potentially exacerbating conflicts within marriages as partners set unattainably high expectations for each other. Conversely, cultures with more flexible standards of success may allow individuals to adopt a more forgiving stance, mitigating some of the relational stress associated with perfectionism.

Additionally, socioeconomic status can influence the impact of perfectionism on marital outcomes. Couples with access to more resources, whether financial, educational, or social, often have greater support networks and tools for managing perfectionist tendencies, potentially reducing the strain on their relationships. In contrast, those with fewer resources may experience a heightened impact from perfectionistic expectations, leading to greater relational dissatisfaction.

Relationship duration is a notable factor influencing the impact of perfectionism on marital outcomes. Studies included in the review suggest that couples with longer relationship histories are more resilient to the negative effects of perfectionism due to developed communication skills and established conflict resolution patterns. Specifically, Vangeel et al. (2020) found that longer durations correlate with reduced marital burnout, as couples better understand each other’s strengths and limitations, which may help manage perfectionistic expectations.

Education level also plays a critical role in moderating perfectionism’s effects on marriage. Individuals with higher educational backgrounds generally have more access to coping resources and support systems, which can buffer against marital strain from perfectionistic expectations. As seen in studies such as Sherry et al. (2014), education can contribute to healthier adaptive perfectionism, which tends to improve relational outcomes by promoting mutual respect and balanced expectations within marriage.

Age range is another influential demographic factor. Younger couples may experience stronger impacts from perfectionism on marital satisfaction due to limited relationship experience, whereas older couples, with greater emotional maturity and experience, can often better manage or moderate perfectionistic tendencies. Research by Minoosepehr et al. (2022) supports this finding, as older couples reported lower levels of marital conflict associated with perfectionism than their younger counterparts.

### How does perfectionism influence marital outcomes?

To interpret the findings of this systematic review, it can be said that healthy or adaptive perfectionism includes aspects of perfectionism related to idealistic efforts, having high personal standards, setting precise criteria for performance, and striving for excellence. Individuals with this type of perfectionism accept personal and situational limitations, challenge themselves, and at the same time have rational goals that allow them to participate in activities and enjoy their successes. In fact, a partner with adaptive perfectionism, whose initial expectations are more flexible, tolerant, and forgiving, may be more inclined to accept that the other partner can perform tasks differently. As a result, individuals with adaptive perfectionism, due to their flexibility and positive emotions, enjoy their marital life more (Flett et al., 1991).

In contrast, maladaptive or negative perfectionism is related to idealistic concerns such as worrying about mistakes, doubts about one’s actions, fear of disapproval from others, and a lack of alignment between expectations and outcomes. This type of perfectionism is positively correlated with maladaptive indicators, such as negative emotions (Harris et al., 2008). Negative perfectionists expect themselves, their life partners, and family members to be perfect, but this unrealistic expectation is not met. As a result, they constantly encounter difficulties in their relationships with their spouses, and trust and friendship in their marital relationships decrease. Negative perfectionists are punctilious, and this behavior diminishes their partner’s self-confidence and becomes distressing for them. This maladaptive pattern of perfectionism leads to marital problems and dissatisfaction in marital relationships (Ehteshamzadeh et al., 2009). Ultimately, due to experiencing a high degree of negative emotions and inhibition in the progression of their marital relationships, maladaptive perfectionists experience a lower level of marital satisfaction.

In support of this explanation, Zuroff and Fitzpatrick (1995) also state that perfectionism regarding interpersonal relationships has a unique ambivalence, consisting of a combination of seeking approval and respect in relationships while simultaneously avoiding relationships due to fear of criticism, control, or humiliation. They have expressed that perfectionists see relationships as means of defining their identity and increasing self-esteem, rather than opportunities for mutual intimacy. Therefore, with an increase in a perfectionist’s distress, they may become more distant interpersonally, which can be evident that the more a person becomes increasingly perfectionistic, their satisfaction with their relationship and life partner decreases.

### Limitations

There were some limitations in the current systematic review. First, the participants of the studies included in the systematic review were only heterosexual couples. Second, there were only two studies in which the relationship between dyadic perfectionism and marital outcomes was examined. Therefore, we were not able to run a separate meta-analysis to gain an effect size for this relationship. Third, there were only two studies in which the relationship between adaptive perfectionism and marital outcomes was examined. Hence, we were not able to run a separate meta-analysis to gain an effect size for the mentioned relationship. Forth, as there were not enough studies to examine the relationship between perfectionism and a specific marital outcome (like marital satisfaction) we were not able to differentiate a particular relationship in our systematic review and meta-analysis. Fifth, the included studies only examined the relationship between perfectionism and marital outcomes and did not provide information regarding the potential role of counseling interventions in preventing or alleviating symptoms. Based on the mentioned limitations, we recommend researchers working in this field of inquiry to conduct a number of studies which consider the relationship between dyadic perfectionism and adaptive perfectionism with the same marital outcome to gain a clear idea regarding this relationship.

### Implications of this systematic review

Based on the results obtained in this systematic review, which indicate that perfectionism plays a significant role in predicting marital relationships, the application of effective therapeutic methods for treating perfectionism is recommended. Accordingly, these therapeutic methods can be provided both before and after marriage. Couples intending to get married are advised to be familiar with adaptive and maladaptive perfectionism, and psychologists are encouraged to explain the difference between these two types of perfectionism to the couples in premarital sessions. This way, the couples become aware that maladaptive perfectionists are likely to have fewer satisfying relationships, while adaptive perfectionists are more likely to have more compatible relationships and higher satisfaction. Additionally, psychologists in these sessions can clarify how the fear of intimacy and avoidant behaviors associated with feelings of inadequacy can weaken the quality of a relationship. Similarly, as criticism of oneself and others is a part of maladaptive perfectionism (Hewitt and Flett, 1991), psychologists can guide couples with maladaptive perfectionism toward constructive relationships.

In the post-marriage phase, perfectionism appears to be a personality trait that needs attention in couples’ therapy, as it can be beneficial in dealing with marital issues. Specifically, focusing on clear self-awareness and understanding of expectations and how these expectations are formed can be helpful. As Weeks and Treat (1992) pointed out, perfectionistic spouses or partners believe that there is a perfect or correct solution for every problem, and they must find it, otherwise the results will be disastrous. In reality, there is no perfect solution, and the inclination toward perfectionism prevents them from recognizing alternative solutions. Such couples lack the ability to generate alternative or creative solutions and believe that the right answer must exist somewhere. Therefore, one of the treatment goals when working with these couples can be to reduce perfectionists’ efforts to achieve personal power and assist them in finding more appropriate and beneficial ways to cope.

One of the treatments that help reduce perfectionism is Enhanced Cognitive-Behavioral Therapy (ECBT) (Fairburn et al., 2008), which is derived from Cognitive-Behavioral Therapy, one of the most widely used forms of psychotherapy. This approach is based on two fundamental principles. First, individuals’ schemas have a controlling effect on their behavior, and second, individuals’ behavior has a significant impact on the cognitive patterns that, when individuals change their behavioral patterns, environmental feedback will change their cognitive patterns (Beck, 2011). Recently, a study has also applied this treatment for perfectionism in students and demonstrated its effectiveness in reducing perfectionism (Hadian Hamedani et al., 2023), suggesting that this treatment can also be used for married individuals. EBCT is designed to help couples identify problematic patterns in their relationship, improve communication and problem-solving skills, and address stress factors arising from within (e.g., infidelity) or external (e.g., job loss). Specifically, EBCT emphasizes helping couples not only address problematic patterns, but also cultivate healthy patterns to foster a stronger and more intimate relationship (Epstein and Zheng, 2017). Therefore, based on the research by Hadian Hamedani et al. (2023) EBCT might be used to assist with perfectionism in married individuals as well.

## Data Availability

The raw data supporting the conclusions of this article will be made available by the authors, without undue reservation.
